# The effects of Src tyrosine kinase inhibitor, saracatinib, on the markers of epileptogenesis in a mixed-sex cohort of adult rats in the kainic acid model of epilepsy

**DOI:** 10.3389/fnmol.2023.1294514

**Published:** 2023-11-09

**Authors:** Nikhil S. Rao, Marson Putra, Christina Meyer, Aida Almanza, Thimmasettappa Thippeswamy

**Affiliations:** Department of Biomedical Sciences, College of Veterinary Medicine, Iowa State University, Ames, IA, United States

**Keywords:** status epilepticus, reactive gliosis, neurodegeneration, microglia morphology, CD68 correlation, glial scars

## Abstract

Neurodegeneration and neuroinflammation are key processes of epileptogenesis in temporal lobe epilepsy (TLE). A considerable number (∼30%) of patients with epilepsy are resistant to currently available antiseizure drugs and thus there is a need to develop adjunct therapies to modify disease progression. A vast majority of interventional strategies to treat TLE have utilized males which limits the translational nature of the studies. In this study, we investigated the effects of repeated low-dose kainic acid (KA) injection on the initial *status epilepticus* (SE) and the effects of Src kinase inhibitor, saracatinib (SAR/AZD0530; 20 mg/kg, oral, daily for 7 days), in a mixed-sex cohort of adult Sprague Dawley rats during early epileptogenesis. There were no sex differences in response to KA-induced SE, and neither did the stage of estrus influence SE severity. KA-induced SE caused significant astrogliosis and microgliosis across the hippocampus, piriform cortex, and amygdala. SAR treatment resulted in a significant reduction of microgliosis across brain regions. Microglial morphometrics such as branch length and the endpoints strongly correlated with CD68 expression in the vehicle-treated group but not in the SAR-treated group, indicating mitigation by SAR. KA-induced SE caused significant neuronal loss, including parvalbumin-positive inhibitory neurons, in both vehicle (VEH) and SAR-treated groups. SAR treatment significantly mitigated FJB-positive neuronal counts as compared to the VEH group. There was an increase in C3-positive reactive astrocytes in the VEH-treated group, and SAR treatment significantly reduced the increase in the piriform cortex. C3-positive astrogliosis significantly correlated with CD68 expression in the amygdala (AMY) of VEH-treated rats, and SAR treatment mitigated this relationship. There was a significant increase of pSrc(Y419)-positive microglia in both KA-treated groups with a statistically insignificant reduction by SAR. KA-induced SE caused the development of classical glial scars in the piriform cortex (PIR) in both KA-treated groups, while SAR treatment led to a 42.17% reduction in the size of glial scars. We did not observe sex differences in any of the parameters in this study. SAR, at the dose tested in the rat kainate model for a week in this study mitigated some of the markers of epileptogenesis in both sexes.

## 1. Introduction

Epilepsy is one of the most common neurological disorders affecting nearly 50 million people globally (WHO, 2023). In the United States alone, there are approximately 3.5 million active cases of epilepsy which is a significant financial burden on the US economy (Begley et al., 2000; Begley and Durgin, 2015; CDC, 2022). Partial onset epilepsy represents roughly 60% of all epilepsies of which temporal lobe epilepsy (TLE) is the most common (Wiebe, 2000; Téllez-Zenteno and Hernández-Ronquillo, 2011). A third of patients with epilepsy are refractory to currently available antiseizure drugs (Kwan and Brodie, 2000; Löscher et al., 2020), prompting a quest for the discovery of novel therapeutic agents targeting alternative pathways. The currently available antiseizure drugs are targeted toward controlling neuronal hyperexcitability through modification of ion channel permeability (Rogawski and Löscher, 2004), and limited effects on the disease onset or its progression (Löscher and Schmidt, 2006).

Neurodegeneration, neuroinflammation, and oxidative stress are well-established markers of epileptogenic changes observed in the brain after a prolonged status epilepticus (SE), the trigger in most cases of acquired epilepsy. Targeting these markers and the mechanism of epileptogenesis is an emerging strategy for disease modification (Löscher and Schmidt, 2006; Löscher et al., 2020). Among several pathways, targeting the Src family kinases (SFKs) has been our area of interest. SFKs are ubiquitously expressed across a variety of cell types in various organs including the nervous system, where they are involved in signaling pathways ranging from neuronal plasticity to microglial proliferation, differentiation, and migration (Purcell and Carew, 2003; Parsons and Parsons, 2004; Portugal et al., 2022). In particular, Fyn, a member of the SFK, has been implicated in mediating neuronal hyperexcitability and microglial activation (Ittner et al., 2010; Panicker et al., 2015). In neurons, Fyn mediates neuronal hyperexcitability via its interaction with tau, leading to tau phosphorylation at Tyr-18, enabling its localization to the dendritic spine where it modulates N-methyl D-aspartate (NMDA) receptor opening and subsequent glutamatergic neurotoxicity (Lee et al., 2004; Ittner et al., 2010; Putra et al., 2020b). There is also evidence to show that phosphorylated Fyn negatively regulates GABAergic receptor expression (Lu et al., 1999; Jurd et al., 2010), potentially reducing inhibitory post-synaptic transmission. We and others have previously demonstrated the role of Fyn-PKCδ signaling in animal models of neurodegenerative disorders including epilepsy (Nygaard et al., 2015; Panicker et al., 2015; Sharma et al., 2018a). In microglia, the Fyn-PKCδ pathway leads to translocation of NFkB via the mitogen-activated protein kinase pathway (MAPK), thereby driving the transcription of proinflammatory cytokines, iNOS and NADPH oxidase (NOX) (Panicker et al., 2015; Sharma et al., 2018a). Therefore, our hypothesis was that pharmacological inhibition of SFKs in neurons and glia present an avenue for neuroprotective roles.

We have previously demonstrated the effects of Fyn/SFK inhibition using saracatinib (SAR; AZD0530), a selective pharmacological inhibitor of SFK/Fyn in kainic acid (KA) and diisopropylfluorophosphate (DFP) models of epilepsy (Sharma et al., 2018a,2021a; Gage et al., 2021a,2022b). SAR is blood-brain-barrier permeable and an excellent orally active investigational test drug (Sharma et al., 2018a). In studies with SAR at 25 mg/kg (Selleckchem source, oral, twice a day for the first 3 days followed by daily single dose for next 4 days) in the KA rat model of TLE, there was a significant reduction in spontaneous seizures, neurodegeneration and markers of oxidative and nitro oxidative stress at 8 days and 3 months post-SE (Sharma et al., 2021a). In a subsequent study with SAR at 25 mg/kg (AstraZeneca, United Kingdom) with a similar dosing regimen in the rat DFP-induced epilepsy model, we noted significant weight loss in the SAR-treated animals versus the vehicle-treated group (Gage et al., 2021a). However, with SAR at 20 mg/kg (AstraZeneca, oral) once-a-day regimen for 7 days in the DFP model there was no significant weight loss and the dose was well tolerated (Gage et al., 2022b). Therefore, in this KA model, a similar dosing regimen of SAR (from AstraZeneca) was followed.

Sex is an important biological variable in translational biomedical research, and thus in 2014, the NIH mandated factoring of both sexes in experimental designs (Clayton and Collins, 2014). In females, typically, the influence of hormones may be manifested in epilepsy as catamenial epilepsy, where there is a cyclical exacerbation of epileptic seizures in relation to altered hormonal levels (Herzog, 2015). Therefore, it is important to consider the influences of sex on treatment outcomes in investigational new drug studies. In this study, we investigated the effects of Fyn/SFK inhibition during the epileptogenic period using SAR at 20 mg/kg (AstraZeneca, oral) in a mixed-sex cohort of young adult rats in the KA model of TLE. We examined sex differences in response to SAR treatment across parameters for neurodegeneration and neuroinflammation, which was not tested in our previous studies in the KA model.

## 2. Methods and materials

### 2.1. Animal studies and ethics statement

35 young adult male (17) and female (18) Sprague Dawley rats (7–8 weeks old) were used in this study. The animals were procured from Charles River (Wilmington, MA, United States) and maintained in the Laboratory of Animal Resources at Iowa State University (ISU). Animals were single-housed in a controlled environment (19°C–23°C, 12 h light: 12 h dark), with unlimited access to food and water. Three days post-acclimatization, the animals were randomized and challenged with kainic acid. All experiments were conducted in accordance with the Institutional Animal Care and Use Committee as per the approved protocols (IACUC-18-159). At the end of each experiment, all animals were euthanized with 100 mg/kg pentobarbital sodium (i.p.) as per the American Veterinary Medical Associations Guidelines for Euthanasia.

### 2.2. Chemicals and reagents

Kainic acid hydrate was procured from Cayman Chemical Company (Ann Arbor, MI, USA). Kainic acid was prepared in sterile water at 5 mg/ml concentration. Saracatinib [SAR/AZD0530; 99.90% pure (RP-UHPLC-MS)] was supplied by AstraZeneca (USA/UK) under the Open Innovation Program. The SAR was dissolved at a concentration of 5 mg/ml in a vehicle comprised of 0.5% hydroxypropyl methylcellulose (HPMC) and 0.1% tween 80 as previously described (Gage et al., 2021a; Sharma et al., 2021a). Diazepam (DZP) was obtained from the Pharmacy of ISU Lloyd Veterinary Medical Center Hospital.

### 2.3. Vaginal cytology and estrus staging

Vaginal lavage for cytology was performed in female rats an hour prior to challenge with kainic acid. The sampling protocols are described in our previous publications (Gage et al., 2020, 2021b). Briefly, using a sterile transfer pipette, approximately 300 μL of sterile normal saline was flushed into the vagina thrice before aspirating it and transferring the lavage contents onto clean chrome alum gelatin-coated slides. The slides were left to dry at room temperature before being stained with 0.1% cresyl violet for a minute followed by washing twice with distilled water. The slides were left to dry before being imaged at 10× magnification under a brightfield microscope (Leica DMi8 with Leica K5 sCMOS camera). The estrus staging was done based on the standardized criteria as published previously (Gage et al., 2020, 2021b). Proestrus was characterized by round, nucleated epithelial cells; cornified, non-nucleated cells with absence of leukocytes in estrus; infiltration of leukocytes along with cornified cells in metestrus; and abundance of leukocytes with or without scant epithelial cells in diestrus.

### 2.4. Kainic acid exposure, treatment, and experimental groups

**FIGURE 1:**
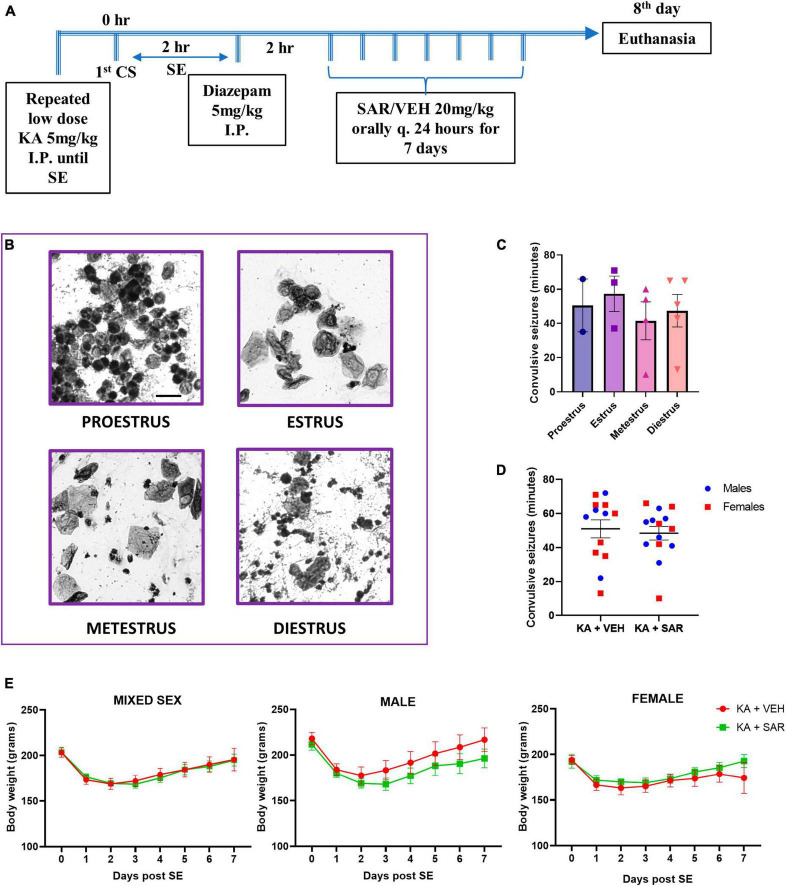
Experimental design. estrus staging, SE severity and body weight progression. **(A)** Animals were challenged with repeated low-dose KA injections until the establishment of SE. Two hours post the first CS, diazepam was administered to terminate SE. Two hours later, SAR or VEH was administered once daily for 7 days. **(B)** Representative cytology of different estrus cycle stages. Scale bar 25 μm. **(C)** The stage of estrus did not have a significant impact on the initial SE severity. **(D)** VEH and SAR-treated animals had the same initial SE severity scores. **(E)** There were no significant differences in the body weight progression between vehicle and SAR-treated animals post-KA challenge. **(C)** One-way ANOVA (Holm-Sidak’s multiple comparisons test), *n* = 2–5; **(D)** two-way ANOVA with Tukey’s multiple comparisons test, *n* = 13–14; **(E)** Mixed-effects analysis, *n* = 12–13. **(C,D)** Data represented as mean ± SEM.

The experimental design is illustrated in Figure 1. We used the repeated low-dose intra-peritoneal protocol for KA injections (5 mg/kg) at 30–40 min intervals not exceeding 30 mg/kg per animal until the establishment of status epilepticus (SE), as previously described (Puttachary et al., 2016; Sharma et al., 2021a). We administered DZP (5 mg/kg, i.p.) 2 h after the onset of the first convulsive seizure, during which time Racine scoring was performed based on our modified Racine scale for chemoconvulsants as published previously (Puttachary et al., 2016; Sharma et al., 2021a; Rao et al., 2022). Animals were randomized again into treatment groups based on matching SE severity. SAR (20 mg/kg, oral) or vehicle was administered 2 h post-DZP, followed by daily dosing for 7 days. Twenty-fours hours after the last dose, the animals were euthanized, and *trans*-cardiac perfusion was done with 4% paraformaldehyde solution. The brains were dissected and processed for immunohistochemical (IHC) analysis.

### 2.5. Initial SE severity and seizure scoring

Behavioral seizures were scored by direct observation of the animals by two experimenters. Video recording of the Racine scoring was also carried out as a backup and also to cross-verify the data. The total number of minutes the animal spent in convulsive seizure stages (≥ stage 3) between the first onset of CS and the time of diazepam injection was calculated to be the SE severity score in minutes. Seizures were scored based on a modified Racine scale (Racine, 1972; Puttachary et al., 2016; Sharma et al., 2021a; Rao et al., 2022). Briefly, stage 1 was characterized by freezing and immobility; stage 2—wet dog shakes, head nodding and facial automatism; stage 3—rearing with forelimb clonus without falling; stage 4—repeated rearing and falling; stage 5—generalized tonic-clonic convulsions with loss of righting reflex, violent jumping/wild running (Supplementary Figure 1A). Only animals having seizure severity score greater than 30 min were included in the IHC data analysis (Guidelines for Epidemiologic Studies on Epilepsy, 1993).

### 2.6. Tissue processing, immunohistochemistry (IHC), imaging and cell quantification

After *trans*-cardiac perfusion and dissection, the brains were fixed in 4% paraformaldehyde for 24 h followed by cryoprotection with 25% sucrose in 0.1 M phosphate-buffered saline (PBS) for another 72 h at 4°C. The brains were then embedded in a gelatin matrix (porcine gelatin type—A, Sigma Aldrich), flash frozen in isopentane supercooled with liquid nitrogen, and tissue blocks were stored at −80°C until sectioning. Brains were cut at 16 μm coronally using a cryostat (ThermoFisher, United States) and mounted over a glass slide coated with chrome-alum gelatin. Starting from the hippocampus, five coronal brain sections were mounted on each slide rostral to caudal such that the distance between brain sections was approximately 480 μm, representing different rostral-caudal aspects of the brain on each slide (Puttachary et al., 2016). The slides were archived and stored at −20°C before processing for IHC. For IHC, antigen retrieval was done in citric acid buffer (10 mM citric acid, 0.05% tween 20, pH 6.0) heated to 95°C for 20 min. Post antigen retrieval, slides were mounted on Shandon racks and washed with PBS, followed by blocking with 10% normal donkey serum for 1 h at room temperature before incubating with primary antibodies overnight at 4°C. The slides were further washed with PBS followed by incubation for an hour with a species-specific fluorophore conjugated/biotinylated secondary antibody at room temperature. Further washing with PBS was done before mounting with Vectashield^®^ anti-fade medium with or without DAPI. Details of primary and secondary antibodies used are listed in Supplementary Table 1.

To evaluate neurodegeneration, NeuN-stained sections were further counterstained with fluoro-jade B (FJB). Briefly, NeuN-stained sections were treated with 0.006% KMnO4 for 5 min followed by three washes with distilled water, 1 min apart. This was followed by incubation with 0.0003% FJB-0.1% acetic acid solution for 10 min in the dark. The slides were allowed to dry before mounting with Acrytol^®^ (Surgipath, Leica Biosystems, IL, USA) hard-setting media. Lecia DMi8 inverted fluorescence microscope equipped with K5 passive cooling sCMOS camera was used for image acquisition.

We maintained consistency in the regions imaged across all the animals, to ensure uniform spatial comparison in the regions of brain pathology across all brains. We analyzed regions of the hippocampus (mesial temporal lobe) such as the dentate gyrus (DG), CA1, CA3 and subiculum (SUB); piriform cortex (PIR) and amygdala (AMY) (neocortical temporal lobe). A minimum of three brain sections per animal were used for cell counting and image analysis as described previously (Sharma et al., 2018b; Putra et al., 2020c). Experimenters were blinded to the experimental groups in all analysis. All images were acquired at 20× magnification unless otherwise specified. ImageJ software was used to quantify cells. pSrc positive microglia, FJB-positive neurons and C3-positive astrocytes were manually quantified using the multipoint tool in Image J as previously described (Putra et al., 2020c; Vasanthi et al., 2023). CD68 colocalization with IBA1 and total IBA1 counts were obtained using the analyze particles function in ImageJ as described previously (Vasanthi et al., 2023). Absolute astrocyte (GFAP-positive cells) counts were obtained using Cell Profiler software (automated quantification) as published previously (Gage et al., 2021b). The percentage area of NeuN-positive staining was used to quantify neuronal loss in different brain regions. The area of glial scars was measured by acquiring lower magnification 4× images of the PIR and AMY regions and tracing the boundary of the scar in ImageJ and averaging the area counts across the sections.

### 2.7. Microglial morphometric analysis

Morphometric analysis of microglia was performed on skeletonized images of IBA1-positive cells from the CA3 region of the brain using previously published protocols (Young and Morrison, 2018; Putra et al., 2020b). Briefly, a 16 μm Z-stack image (10 images per stack) of IBA1-positive cells from the CA3 region of the hippocampus was captured at 20× magnification from 2 sections per animal. This enabled us to capture details of microglial processes from multiple focal planes. We used the Maximum Contrast Projection package in R^1^ to render 2D images from the Z-stacks. Further processing of the images was carried out using Image J. A 300 μm × 300 μm field of the images was cropped out for further analysis. The images were binarized and skeletonized before running the Analyze Skeleton plugin^2^ to generate data related to the number of branches, number of endpoint voxels and average branch length. The area of the microglial cell body was quantified using the analyze particles function in Image J on a thresholded image of microglial cell bodies. All morphometric parameter values were divided by the total number of cells in the field to yield average numbers per cell for final analysis.

### 2.8. Experimental design, rigor, and statistical analysis

All experiments were subject to rigorous experimental methodology and blinding where appropriate. Animals were randomized into treatment groups based on matching SE severity. This was done to ensure the distribution of animals with similar brain pathologies across the groups. GraphPad Prism 9.0 was used for statistical analysis of the datasets. The normality of the data was analyzed using the Shapiro–Wilk test, whenever applicable. While comparing two groups, we used the *t*-test or Mann–Whitney test depending on the normality of the data. One-way ANOVA with Tukey’s *post-hoc* test was used to compare multiple groups with one factor, while sex differences were tested using the two-way ANOVA to test for significant interactions between sex and treatment effects (Garcia-Sifuentes and Maney, 2021). If there were no significant sex interactions, then a repeated measures two-way ANOVA or mixed-effects model with Tukey’s multiple comparisons test was used to compare regional differences. Bar graphs were plotted representing the mean ± SEM with individual datapoints. A mixed-effects model or repeated measures two-way ANOVA was performed to determine the overall main treatment effects when comparing across different brain regions in immunohistochemistry. To show the overall effect, the data was presented as a boxplot representing the median, quartiles, minimum and maximum values of the data distribution. We used the Spearman correlation coefficient for correlational analysis, and slope comparisons were made using simple linear regression. Detailed statistical analysis is described in the figure legends. The *p*-values of the region-wise two-way ANOVA interaction test for sex differences are listed in Supplementary Table 2.

## 3. Results

### 3.1. Initial SE severity comparison between vehicle and SAR-treated groups, the effects of estrus stages on SE severity, and the effect of KA and SAR on body weight

An overview of the experimental design is illustrated in Figure 1A. Representative images of vaginal cytology from different estrus stages are shown in Figure 1B. There were no significant differences in the SE severity scores in different stages of estrus (Figure 1C). Vehicle and SAR-treated animals had similar initial SE severity scores, and neither was there any influence of sex in response to KA-induced SE (Figure 1D). There was no significant difference in the number of KA doses required to induce SE between male and female animals, and neither was there a difference in the latency to the onset of convulsive seizure following the last KA injection (Supplementary Figures 1B, C). Six percent animals died during SE. Three animals that had SE severity scores less than 30 min were excluded from IHC analysis. There were no significant differences in the body weight recovery between the VEH and SAR-treated rats in either sex after the KA challenge (Figure 1E).

### 3.2. SAR treatment significantly reduced microgliosis at 8 days post-SE

**FIGURE 2:**
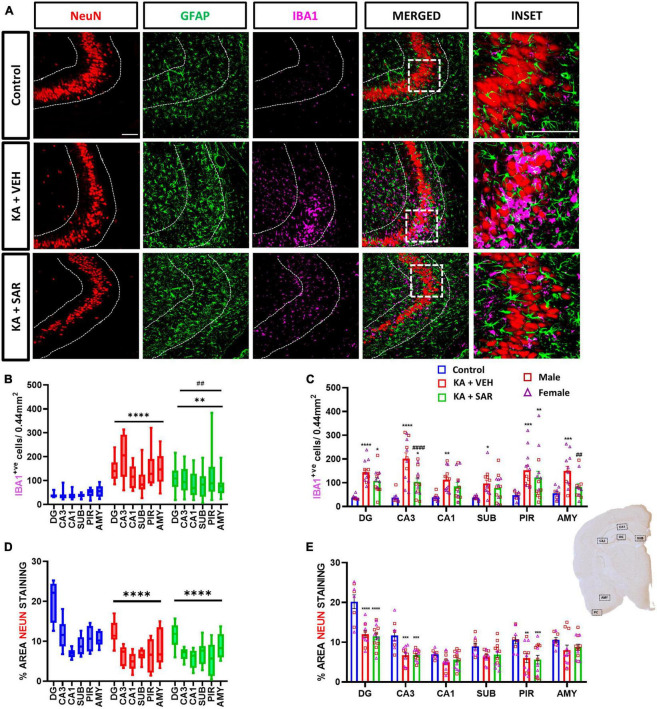
Microgliosis and neuronal loss. **(A)** Representative images of the CA3 region of the hippocampus of different treatment groups stained for NeuN (neurons), GFAP (astrocytes) and IBA1 (microglia). Scale bar 100 μm. **(B)** SAR treatment significantly attenuated overall SE-induced microgliosis in the brain at 8 days post-SE. **(C)** Microgliosis in the hippocampal regions (DG, CA3, CA1, SUB), piriform cortex (PIR) and amygdala (AMY). **(D)** SAR treatment did not rescue SE-induced neuronal loss. **(E)** NeuN staining in the hippocampal regions (DG, CA3, CA1, SUB), PIR and AMY. **(B–E)** Mixed-effects analysis (Tukey’s multiple comparisons test) *n* = 8–14. *p < 0.05, **p < 0.01, ***p < 0.001, ****p < 0.0001 vs. control; ##*p* < 0.01, ####*p* < 0.0001 vs. KA+VEH. **(C,E)** Data represented as mean ± SEM.

Representative images of the CA3 region of the brain stained for NeuN, GFAP and IBA1 are shown in Figure 2A and Supplementary Figure 2. KA-induced SE caused significant increases in microglia (IBA1-positive cells) in all brain regions of interest in the vehicle-treated group compared to controls (Figure 2C) 8 days post-SE. SAR-treated rats also had a significant increase in microglia cell counts in the DG, CA3 and PIR regions versus the controls (Figure 2C). SAR treatment significantly reduced microgliosis in the CA3 and AMY regions of the brain when compared to vehicle-treated rats. Two-way ANOVA revealed no significant sex and treatment interactions across any of the regions of interest. Overall, in the mixed-effects analysis across all brain regions, we found significant increases in microgliosis in both KA-treated groups versus controls, with a significant reduction in microgliosis in the SAR-treated group versus the KA vehicle group (Figure 2B).

### 3.3. The effects of SAR on SE-induced neuronal loss

**FIGURE 3:**
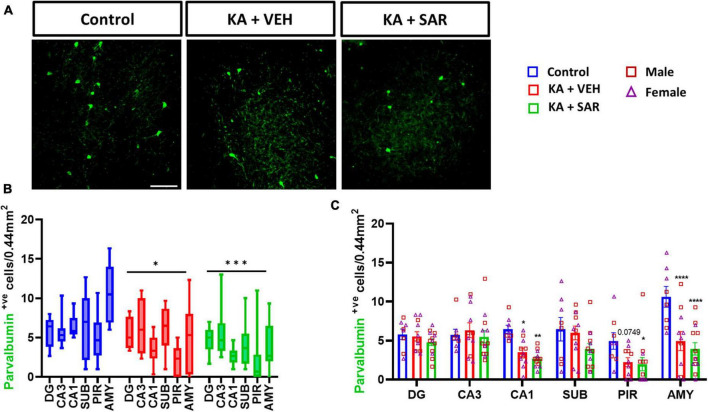
SAR treatment did not rescue SE-induced inhibitory neuronal loss. **(A)** Representative images of inhibitory neurons (PV-positive cells) in the AMY region of the brain from the different treatment groups. Scale bar 100 μm. **(B,C)** Inhibitory neuronal counts in the hippocampal regions (DG, CA3, CA1, SUB), piriform cortex (PIR) and amygdala (AMY). **(B,C)** Repeated measures two-way ANOVA (Tukey’s multiple comparisons test), *n* = 8–14. *p < 0.05, **p < 0.01, ***p < 0.001, ****p < 0.0001 vs. control. **(C)** Data represented as mean ± SEM.

We observed an overall significant reduction of NeuN staining in all regions of the brain in the KA-treated groups versus the controls (Figure 2D), primarily driven by significant reductions of NeuN staining in the DG, CA3 and PIR (Figure 2E). We did not observe significant sex differences in any of the regions of interest (Figure 2E). Histologically, the regions of neuronal loss coincided with regions of reactive microglial infiltration and astrogliosis (Figure 2A, Supplementary Figure 2). SAR had no effect on mitigating the SE-induced neuronal loss. We further examined parvalbumin (PV) positive neurons, a subset of GABAergic inhibitory interneurons. Representative PV staining in the AMY is shown in Figure 3A and Supplementary Figure 3. We noted significant inhibitory neuronal loss in the CA1 and AMY regions of the brain in both KA groups versus the controls (Figure 3C). Interestingly, there was significant PV neuronal loss in the PIR region of SAR treated rats compared to the controls (Figure 3C). No significant sex differences were observed in PV neuronal loss. Overall, we noted significant parvalbumin-positive neuronal loss in the KA groups irrespective of SAR or vehicle treatment (Figure 3B).

### 3.4. SAR treatment significantly reduced KA-induced FJB-positive staining at 8-days post-SE

**FIGURE 4:**
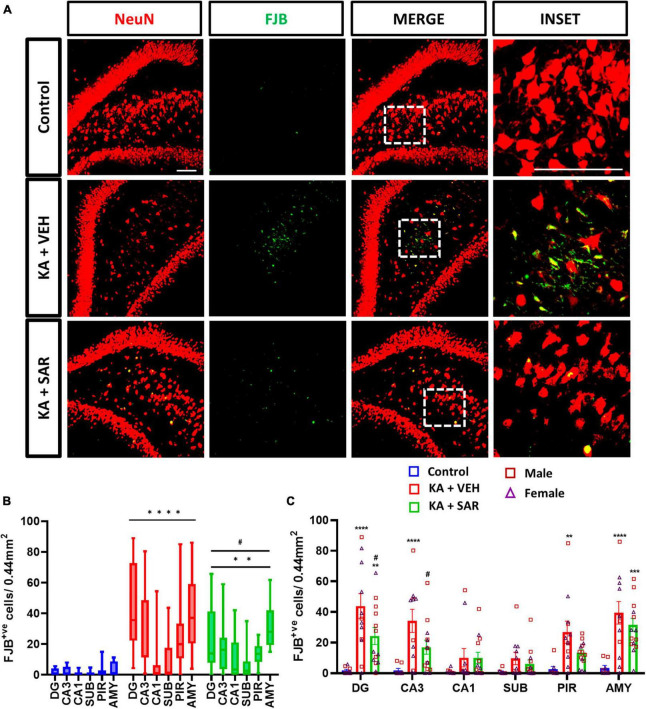
SAR treatment significantly attenuated SE-induced neurodegeneration. **(A)** Representative images of degenerating neurons (FJB positive cells) in the DG region of the hippocampus from the different treatment groups. Scale bar 100 μm. **(B,C)** Neurodegeneration in the hippocampal regions (DG, CA3, CA1, SUB), piriform cortex (PIR) and amygdala (AMY). **(B,C)** Repeated measures two-way ANOVA (Tukey’s multiple comparisons test). *n* = 8–14. **p < 0.01, ***p < 0.001, ****p < 0.0001 vs. control; #*p* < 0.05 vs. KA+VEH. **(C)** Data represented as mean ± SEM.

Representative images of NeuN and FJB stained cells in the DG of the hippocampus are shown in Figure 4A and Supplementary Figure 4. FJB staining was used as a marker for degenerating neurons. There were significant increases in FJB-positive cells in the DG, CA3, PIR and AMY regions of the brain in the VEH-treated group versus the control group (Figure 4C). SAR significantly reduced KA-induced neurodegeneration in the DG and CA3 regions of the hippocampus compared to the VEH group (Figure 4C). We did not observe sex differences in neurodegeneration in any of the regions. Overall, there was a significant increase in neurodegeneration across all regions in both KA groups, with significant mitigation by SAR (Figure 4B).

### 3.5. SAR significantly reduced KA-induced C3 positive astrocytes in the piriform cortex but not in the other regions

**FIGURE 5:**
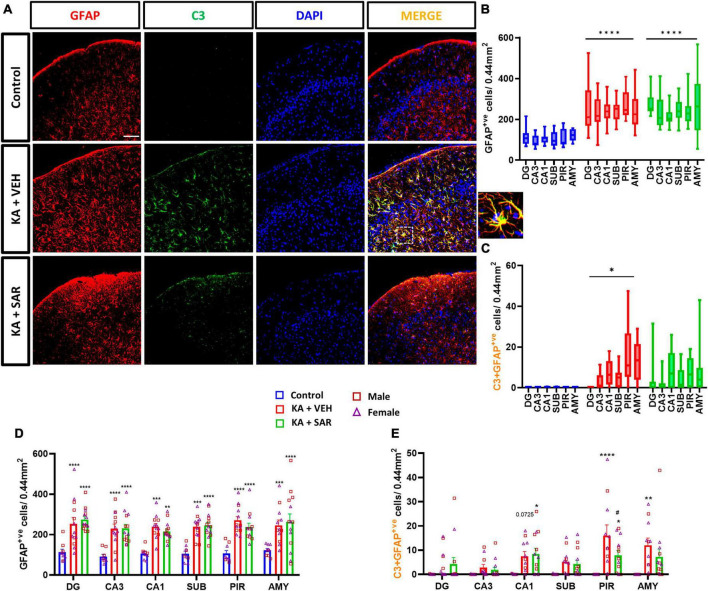
Astrogliosis and reactive astrogliosis. **(A)** Representative images of astrocytes (GFAP positive cells) and reactive astrocytes (C3+GFAP colocalized cells) in the PIR region of the different treatment groups. An enlarged image of a C3-positive astrocyte is shown. Scale bar 100 μm. **(B,D)** SAR treatment did not have an effect on SE-induced astroglial proliferation in the hippocampal regions (DG, CA3, CA1, SUB), piriform cortex (PIR) and amygdala (AMY). **(C)** SE led to an overall increase in reactive astrocytes in the VEH-treated animals, but not in the SAR-treated group. **(E)** SAR mitigated SE-induced reactive astrogliosis in the PIR. **(B–E)** Mixed-effects analysis (Tukey’s multiple comparisons test) *n* = 8–14. *p < 0.05, **p < 0.01, ***p < 0.001, ****p < 0.0001 vs. control; #*p* < 0.0001 vs. KA+VEH. **(D,E)** Data represented as mean ± SEM.

Representative images of the PIR region immunostained for GFAP and C3 are shown in Figure 5A and Supplementary Figure 5. We noted significant increases in astroglia (GFAP-positive cells) across all brain regions irrespective of SAR or VEH treatment in the KA-treated animals. No significant sex differences were detected in the region-wise two-way ANOVA interaction tests (Figure 5D). There was an overall significant increase in astrogliosis in the mixed-effects analysis across the regions (Figure 5B). We used the complement 3 (C3) as a marker for reactive astrocytes (Liddelow and Barres, 2017; Putra et al., 2020a). There were significant increases in reactive astrocytes (C3+GFAP positive cells) in the PIR and AMY regions in the vehicle-treated group versus the controls (Figure 5E). SAR treatment significantly reduced C3-positive astrogliosis in the PIR in contrast to the vehicle-treated group (Figure 5E). We did not find significant sex differences in reactive astrogliosis in any regions. In the mixed-effects analysis for reactive astrocytes, we noted an overall significant increase in the vehicle-treated groups, while SAR treatment decreased the C3-positive astrogliosis by about 25% (Figure 5C).

### 3.6. SAR treatment mitigated the correlation between phagocytic microgliosis and reactive astroglia induced by KA

**FIGURE 6:**
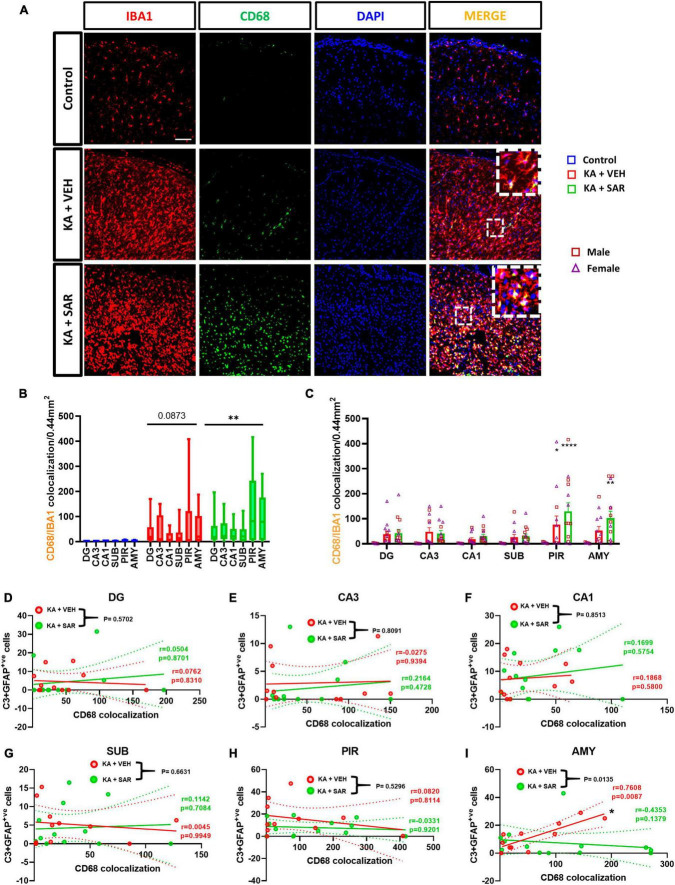
Phagocytic microgliosis and correlation with reactive astrogliosis. **(A)** Representative images of phagocytic microglia (CD68+IBA1 positive cells) in the PIR region of the brain. Scale bar 100 μm. **(B,C)** SAR-treated animals showed a significant overall increase in phagocytic microglia, primarily driven by increases in the PIR and AMY regions of the brain. **(D–I)** There was a significant positive correlation between C3-positive astrogliosis and CD68 expression in the AMY of VEH-treated rats, and SAR treatment significantly uncoupled this relationship. **(B,C)** Repeated measures two-way ANOVA (Tukey’s multiple comparisons test), *n* = 8–14. *p < 0.05, **p < 0.01, ****p < 0.0001 vs. control. **(D-I)** Spearman correlation with simple linear regression for comparison between slopes. The dotted lines represent the 95% confidence intervals for the two means, *n* = 11–13. **(C)** Data represented as mean ± SEM.

CD68 was used as a marker for phagocytic or reactive microglia (Kettenmann et al., 2011). Representative images from the PIR are shown in Figure 6A and Supplementary Figure 6. The KA-VEH group showed significant increases in CD68+IBA1 colocalization only in the PIR (Figure 6C). Interestingly, unexpectedly, we observed significant increases in CD68+IBA1 colocalization in the PIR and AMY regions in SAR treated group (Figure 6C). However, we did not find significant sex differences in phagocytic microgliosis in any of the regions. Overall, KA treatment caused increases in phagocytic microgliosis in both groups across the brain regions versus the controls, with SAR-treated animals showing significant overall increases in CD68+IBA1 colocalization (Figure 6B). We further examined the relationship of CD68-positive microgliosis and C3-positive astrogliosis in the different brain regions (Figures 6D–I). We noted a significant and strong positive correlation (*p* = 0.0087) between CD68 expression and reactive astrogliosis in the AMY region of the brain in the VEH-treated group, and SAR treatment significantly mitigated this effect (*p* = 0.0135) (Figure 6I).

### 3.7. Impact of KA and SAR on reactive microglial morphometric parameters and its correlation with CD68 expression

**FIGURE 7:**
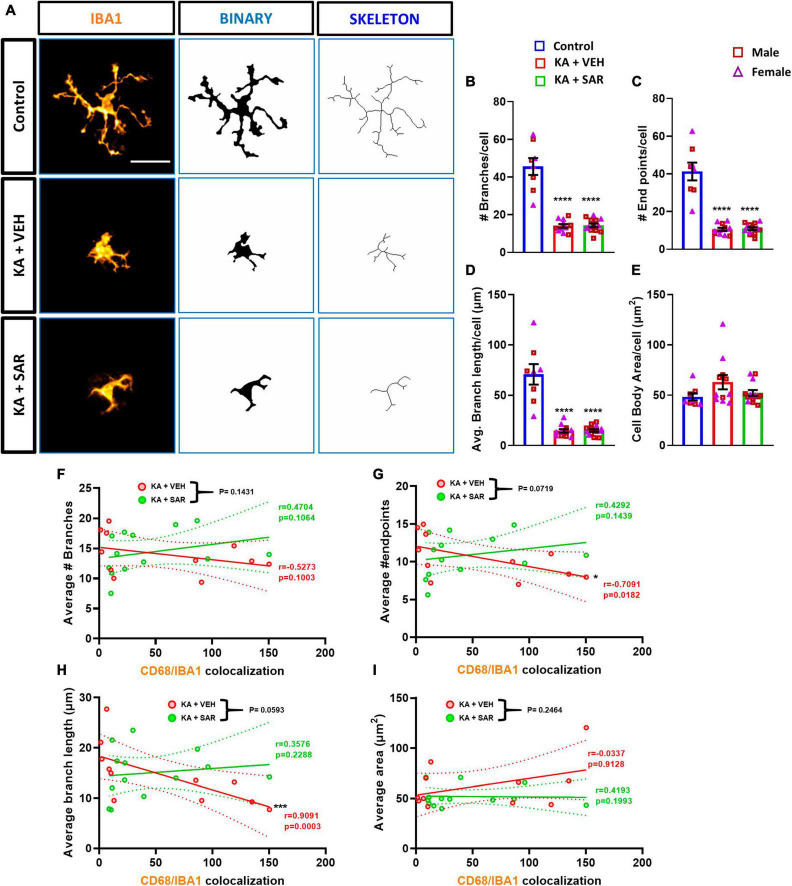
Morphometric analysis of microglia and correlation with CD68 expression. **(A)** Representative images of individual microglia from the CA3 region of different treatment groups along with respective skeletonized images. Scale bar 25 μm. **(B–D)** There was a significant reduction in the number of branches, endpoints, and average branch length of microglia in kainic acid-treated groups. **(E)** There were no significant differences in the area of microglial cell bodies between the treatment groups. **(F–I)** There was a significant negative correlation between the average number of microglial endpoints, average branch length and CD68 expression in the VEH-treated groups but not in the SAR-treated group. **(B–E)** Ordinary one-way ANOVA (Tukey’s multiple comparisons test), *n* = 8–14, ****p < 0.0001 vs. Control; **(F–I)** Spearman correlation with simple linear regression for comparison between slopes. The dotted lines represent the 95% confidence intervals for the two means, *n* = 11–13. *p < 0.05, ***p < 0.001. **(B–E)** Data represented as mean ± SEM.

Representative images of IBA1 cells and corresponding skeletonized images are shown in Figure 7A. We observed a significant reduction in the number of branches, endpoints, and average branch length of microglia in KA-treated groups versus the controls (Figures 7B–D). These results indicate a change in the morphology of microglia from the resting, ramified type to the reactive, amoeboid type. Interestingly, there were no significant differences in the area of the cell bodies between the treatment groups (Figure 7E). These findings indicate that kainic acid-induced the polarization of microglia to the reactive type, and SAR did not prevent microglia polarization. There were no significant sex differences across any of the morphological parameters. We thus further investigated the relationship of microglial morphological parameters with the extent of CD68 expression in the CA3 region of both KA-treated VEH and SAR groups (Figures 7F–I). There was a strong and significant negative correlation between the average number of endpoints (*p* = 0.0182) and the average branch length (*p* = 0.0003) of microglia with the CD68 colocalization counts from the corresponding regions in the vehicle-treated group (Figures 7G, H). SAR treatment mitigated the relationship between microglial morphological state and phagocytic activity (Figures 7G, H). However, we did not observe significant correlations between the number of microglial branches (Figure 7F), microglial cell body area (Figure 7I) and CD68 expression in either of the groups.

### 3.8. The effects of SAR on SE-induced Src(pY419)-positive microglia

**FIGURE 8:**
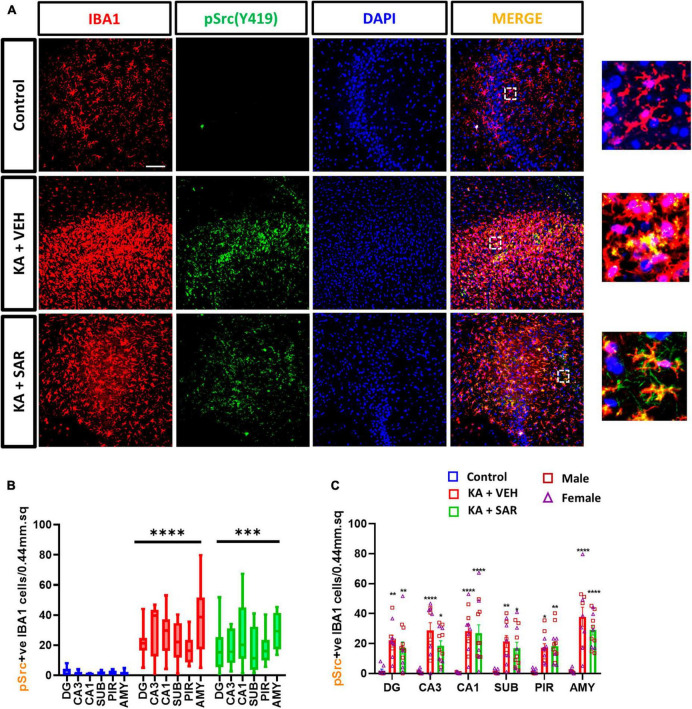
KA-induced SE led to a significant upregulation of pSrc(pY419)-positive microglia. **(A)** Representative images of pSrc-positive microglia (pSrc+IBA1 positive cells) in the CA3 region of the hippocampus from different treatment groups. Scale bar 100 μm. **(B,C)** pSrc-positive microglia in the hippocampal regions (DG, CA3, CA1, SUB), piriform cortex (PIR) and amygdala (AMY). **(B,C)** Mixed-effects analysis (Tukey’s multiple comparisons test), *n* = 8–14. *p < 0.05, **p < 0.01, ***p < 0.001, ****p < 0.0001 vs. control. **(C)** Data represented as mean ± SEM.

The Fyn-PKCδ signaling pathway has been implicated in microglial activation (Panicker et al., 2015; Sharma et al., 2018a). We then quantified the total number of pSrc(pY419) expressing microglia, a marker for activated Fyn/Src, in the brain. Representative images of pSrc-positive microglia from the CA3 region of the hippocampus are shown in Figure 8A and Supplementary Figure 7. We noted a significant increase in pSrc-expressing microglia in both kainic acid-treated groups across all brain regions, and SAR did not reduce its levels (Figures 8B, C).

### 3.9. SAR effects on the KA-induced cortical glial scars

**FIGURE 9:**
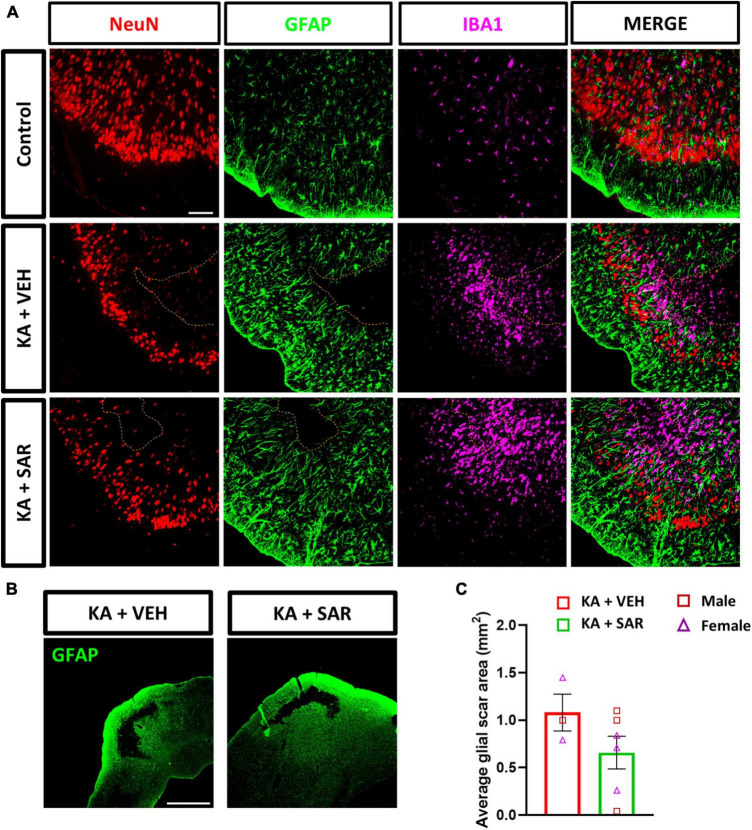
KA-induced SE led to the formation of classical glial scars in the piriform cortex of the brain. **(A)** Representative images of the PIR region showing glial scars (dotted line) in the KA-treated groups versus the similar region of a control animal stained for NeuN (neurons), GFAP (astrocytes) and IBA1 (microglia). Scale bar 100 μm. **(B)** Representative low-magnification images of glial scars in the PIR of KA-treated rats. Scale bar 1,000 μm. **(C)** There was a 42.17% reduction in the size of the glial scar of the SAR-treated group versus the VEH. Unpaired *t*-test, *n* = 3–6. **(C)** Data represented as mean ± SEM.

Previously, we demonstrated the occurrence of classical cortical glial scars in the rat diisopropylfluorophosphate (DFP) model (Gage et al., 2022a; Meyer et al., 2023). In this study we observed classical glial scars in the piriform cortex of some rats at 8 days post KA. Representative images of glial scars co-immunostained for NeuN, GFAP and IBA1 are shown in Figures 9A, B. We observed glial scars in 3 out of 11 rats treated with VEH, and in 6 out of 13 rats in the SAR group. There were no significant predispositions to the occurrence of glial scars in either group (Fisher’s exact test). There was a 42.17% reduction in the size of the glial scars in the SAR-treated group compared to the VEH group, however, the difference was not statistically significant (Figure 9C). Histologically, the scars had a core that was devoid of astrocytes (GFAP-positive cells) and neurons (NeuN-positive cells); while the periphery of the scar was lined with hypertrophic (A1-like) astroglia and the core was infiltrated with reactive microglia (IBA1-positive cells) (Figure 9A).

## 4. Discussion

Kainic acid (KA) has been used to model TLE in rodents for several decades as it recapitulates many of the features of human TLE, including anatomical, neurobehavioral, and electrophysiological features (Rusina et al., 2021). Our present study is a rigorous investigation of SFK inhibition on markers of neuroinflammation and neurodegeneration during early epileptogenesis in the rat KA model of TLE. It also provides a thorough investigation into sex differences across these parameters to decipher the influence of sex hormones on the outcomes of SFK inhibition. We investigated epileptogenesis parameters such as microgliosis, astrogliosis, neuronal loss, neurodegeneration, phagocytic microgliosis and its relationship with microglial morphometrics, inhibitory neuronal loss, and for the first time, reported the occurrence of cortical glial scars, and CD68 correlation with morphometric parameters and reactive astrogliosis in the rat KA model of TLE.

Sex is an important biological variable in translational biomedical research, including epilepsy, since the influence of hormones can modulate seizure, neuropathology and treatment outcomes (Singer et al., 1996; Clayton and Collins, 2014; Scharfman and MacLusky, 2014; Herzog, 2015; Reddy, 2016; Reddy et al., 2019). A large number of studies in epilepsy research have been conducted in male rats; thus extrapolating these findings to female populations might not be straightforward (Rusina et al., 2021). For example, adult female rats are relatively resistant to pilocarpine-induced seizures compared to males, while increased susceptibility to seizures, neurodegeneration and astrogliosis were reported in aged female C57BL/6 mice after KA-induced excitotoxicity compared to their male counterparts (Zhang et al., 2008; Scharfman and MacLusky, 2014). In this study, we used the repeated low-dose method of KA injection to induce SE, and we observed no sex differences in the initial SE severity, indicating that both sexes responded equally to the repeated low-dose KA approach. The repeated low-dose method of KA injection ensures consistent SE severity with low mortality and increases the likelihood of animals becoming epileptic (Puttachary et al., 2015). In the present study, roughly 90% of rats exposed to kainic acid developed convulsive seizures > 30 min, and the three animals that had SE scores less than 30 min were excluded. The stage of the oestrus cycle also did not influence the outcome of SE severity in female rats (Figure 1C), which was similar to our findings from the soman (GD) rat model of epilepsy (Gage et al., 2021b). Traditionally, investigations into sex differences in biomedical research, including epilepsy, have often involved disaggregating data by sex and examining the cohorts separately. Such an analysis may not always yield meaningful interpretations in interventional drug studies (Garcia-Sifuentes and Maney, 2021). In this study, therefore, we used a factorial design to investigate the interaction of sex on treatment outcomes and we did not find differential response of sex on treatment outcomes.

The gold standard to detect spontaneous seizures in animal models is electroencephalography (EEG). However, in this study, the aim was to investigate the efficacy of SAR treatment at 20 mg/kg dosing regimen on neuropathological hallmarks of early epileptogenesis in non-telemetered animals in a short-term study. Telemetry implanted animals were part of another study (Sharma et al., 2021a), which is beyond the scope of the current study. We have previously demonstrated the long-term (4 months post-SE) washout effects of early SAR treatment in both telemetry and non-telemetry experiments (Sharma et al., 2021a). Therefore, in the current mixed sex cohort of short-term study, EEG does not add much to the overall outcome of the study. Furthermore, continuous (24/7) EEG recording is not cost-effective but useful in long-term studies to capture spontaneously recurring seizures, which normally occur a week after the onset of SE (Puttachary et al., 2015; Sharma et al., 2018b,2021a). The present study investigated the effects of SFK inhibition using SAR at 20 mg/kg once a day for 7 days on brain markers of epileptogenesis in a mixed-sex cohort of adult rats. SAR is a potent and highly selective inhibitor of Src family tyrosine kinases (SFKs) (IC_50_ of 2.7–11 nM for Fyn and Src) (Green et al., 2009). In our previous study in the rat KA model, we used SAR orally at 25 mg/kg twice daily for the first 3 days, followed by once a day dosing for the next 4 days (Sharma et al., 2021a). SAR treatment significantly reduced spontaneous seizures and levels of pSrc-Y416 and pPKCδ-Y311 in the 4 months following treatment. However, a major limitation of the study was that it did not factor in sex as a biological variable and a low dose SAR was not tested. Similar to the previous DFP study (Gage et al., 2022b), we noted no significant weight loss between the SAR and VEH-treated groups in this study. An important point to note is that in the previous DFP study and in the present study, the SAR was procured from AstraZeneca, and the LC-MS/MS analysis revealed its purity at > 99%, compared to commercially available SAR which had a purity of ∼90% by LC-MS/MS. Therefore, the higher concentration of the > 99% pure drug from AstraZeneca might have exacerbated the weight loss that was seen in the DFP studies with a 25 mg/kg dosing regimen twice a day during the first 3 days post-SE (Gage et al., 2021a).

Currently available antiseizure therapies are targeted to control seizures and do little to prevent the underlying brain pathology (Löscher and Schmidt, 2006). Neuroinflammation is characterized by microgliosis, astrogliosis and oxidative stress, the pathological hallmarks of epileptogenesis (Vezzani et al., 2016, 2019). Epileptic seizures result from an imbalance in the populations of excitatory and inhibitory neurons (Engel, 1996; Day et al., 2012). Parvalbumin interneurons are a subpopulation of inhibitory neurons that express the calcium-binding protein parvalbumin, and have been extensively studied in epilepsy (Godoy et al., 2022; Juarez and Martínez Cerdeño, 2022). Parvalbumin neurons are critical players in maintaining the excitatory and inhibitory balance in the brain and loss of these neurons can lead to disrupted excitability and increased seizure susceptibility (Sharma et al., 2021b; Godoy et al., 2022). The neurons in the hippocampus are susceptible to neurotoxicity induced by KA because of the high concentrations of AMPA/KA receptors in the region (Zheng et al., 2011; Rusina et al., 2021). Similarly, neurons in the extrahippocampal regions like the piriform cortex and amygdala are also vulnerable to KA-induced excitotoxicity (Pereno and Beltramino, 2009). In our study, we observed a significant loss of neurons including PV-positive inhibitory neurons, in the KA-treated groups due to SE-induced excitotoxicity by KA. SAR did not mitigate the KA-induced neuronal loss. However, SAR significantly mitigated neurodegeneration as indicated by FJB staining. This could be a direct effect of reduced microglial proliferation by SAR. Microglial proliferation occurs during early epileptogenesis to phagocytose degenerating neurons (Di Nunzio et al., 2021). It is possible that the loss of neurons observed in the groups treated with KA may be due to irreversible neuronal injury from the impact of initial SE (Dingledine et al., 2014) and the SAR treatment for a longer duration may be required to rescue neurons, including the inhibitory neurons, as in the rat soman model of epilepsy (Vasanthi et al., 2023). FJB has also been proposed as a marker for activated glia in a transgenic mouse model of Alzheimer’s disease (Damjanac et al., 2007). However, in this study we co-labeled FJB with NeuN to ascertain the degenerating neurons.

In this study, we observed a significant increase in microglial and astroglial proliferation across all brain regions in the KA groups versus the controls. SAR significantly mitigated microgliosis but not astrogliosis. However, SAR did mitigate C3 positive astrocytes (reactive astrogliosis). Astrocytes are involved in potassium and glutamate reuptake from the extracellular space under physiological conditions and after seizure activity. However, the dysfunctional or reactive astrocytes seen in epilepsy, lose their ability to effectively clear excess glutamate and K^+^, further exacerbating neuronal excitation (Coulter and Steinhäuser, 2015). Reactive astrocytes secrete C3 while microglia contain C3 receptors, which leads to their activation (Lian et al., 2016; Wei et al., 2021). In our study, in addition to astrocyte proliferation post-SE, we noted a significant increase in C3-expressing reactive astrocytes in the VEH-treated group. Such an increase was not observed in the SAR-treated group, indicating a protective effect. Reactive microglia secrete complement C1q while astrocytes possess C1q receptors, which can induce A1-like reactive astrocytes (Liddelow et al., 2017). Although we did not examine the expression of C1q in microglia, we studied the expression of CD68 expression in microglia since C3-positive reactive astrocytes have been found to be in close association with CD68-positive microglia in other neurodegenerative diseases such as multiple sclerosis (Liddelow et al., 2017). We therefore examined the relationship between C3-positive astrogliosis and CD68 expression. We noted a significant positive correlation between C3-positive astrocytes and CD68 expression in the AMY of VEH-treated rats and SAR treatment significantly altered this association.

CD68 is a lysosomal transmembrane glycoprotein and a marker for reactive or phagocytic microglia (Kettenmann et al., 2011). Interestingly, in our study, we noted a significant increase in CD68-positive reactive microgliosis in the SAR-treated group versus controls, especially in the extrahippocampal regions. A similar trend of increased CD68 expression was observed in these regions of SAR-treated male rats in the previous DFP study (Gage et al., 2022b). This may appear contradictory to SAR’s protective role reported in previous publications in KA and DFP models (Sharma et al., 2021a; Gage et al., 2022b). However, in this study, the reduction in FJB positive cells, microgliosis, and reactive astrogliosis (C3 positive astrocytes) in SAR treated group imply that the increased CD68 positive microglia may suggest phagocytosing the dead cells and clearing tissue debris to minimize localized toxicity.

Microglia can assume a variety of morphologies based on their phenotypic state of reactivity and broadly categorized as resting or activated (Streit et al., 1999; Wyatt-Johnson et al., 2017; Lier et al., 2021). Manual, morphology-based characterization of reactive microglia may be biased. In this study, we conducted an objective analysis of binarized skeletons of IBA1 cells from the CA3 region of the hippocampus since this was one of the regions where SAR significantly reduced microgliosis. Morphometric analysis of microglia revealed that KA-induced the polarization of microglia to the reactive type, and SAR did not have an effect in preventing polarization, which may be beneficial for clearing the cellular debris. We thus further investigated the relationship of microglial morphometric parameters with the extent of CD68 expression. The significant negative correlation between the average number of endpoints, average branch length and CD68 expression in the vehicle-treated group meant that the more activated and phagocytic the microglia were, the less branched they were, which is consistent with the characteristics of the colloquial, and a controversial, “M1-like” microglia. These correlations are consistent with the idea that microglia change their morphology according to their function (Lawson et al., 1990). We did not observe significant correlations in these parameters of the SAR-treated group, which means that SAR uncoupled this relationship, allowing microglia to be both branched and phagocytic. Whether or not the increased phagocytic activity of microglia aids in the clearing of debris post-SE or contributes to the progression of the disease remains a conundrum (Minett et al., 2016; Wyatt-Johnson and Brewster, 2019). There is a general consensus that microglia can adopt a diverse spectrum of morphologies and functions during different stages of an inflammatory response. A mere categorization of microglia into resting or reactive type based on either morphology or a single biomarker does not capture the entire spectrum of its functional activity; thus there has not been a widely acceptable specific marker of microglial activation (Lier et al., 2021; Paolicelli et al., 2022). A high throughput single-cell sequencing is a versatile tool to study microglial dynamics and can be used to elucidate the microglia-mediated mechanisms in future studies in KA model.

Recently, we demonstrated the occurrence of classical glial scars in the piriform cortex of rats in the DFP model (Gage et al., 2022a; Meyer et al., 2023). One other previous study has reported the occurrence of similar glial scars in the thalamus after intra-thalamic KA injection (Dusart et al., 1991), which could be due to trauma at the injection site. Similar to our previous studies in the DFP model, we found the occurrence of classical glial scars in the piriform cortex of rats after intraperitoneal injection of KA. When we compared the size of the glial scars between the VEH and SAR-treated rats, we noted a reduction in the area of glial scars in the SAR-treated rats, although this was not statistically significant given the low number of animals (*n* = 3) showing glial scars in the VEH group. This could be related to the suppression of reactive astrogliosis by SAR in the PIR (Figure 5E), since severe reactive astrogliosis is frequently associated with glial scar formation (Sofroniew and Vinters, 2010). Interestingly, although we noted extensive pathology in the hippocampus of KA-treated rats, glial scarring was observed only in the piriform cortex, indicating an intrinsic predilection for the development of scars in this region across chemo-convulsant-induced SE models. Based on extensive literature search, cortical glial scars are not yet reported in the KA model of TLE.

SAR is a potent and highly selective inhibitor of Src/Abl kinase (Hennequin et al., 2006; Green et al., 2009). Our previous SAR studies demonstrated reductions in the levels of pSrc from whole-hippocampal lysates at 8 days post-SE with a different dosing regimen (Sharma et al., 2021a). In this study we did not observe a significant reduction in pSrc-positive microglia in the SAR-treated group, although we observed a trend in the reduction in all regions of the brain except the piriform cortex. Moreover, SFKs are also expressed in neurons, which we did not investigate in this study. It is also worth noting that the affinity of SAR for activated Src(pSrc) is ten times higher than for native Src, and inconsistent reductions in the levels of pSrc(Y419) after SAR treatment have been reported (Green et al., 2009). Therefore, while interpreting the pSrc(Y419) changes, the cell types, the region, and the role of other Src tyrosine family members involved in the process should be considered.

In this study, we demonstrated the effects of SAR treatment on neuroinflammation and neurodegeneration markers after KA-induced SE. These limited effects of SAR may not be enough for preventing or modifying all aspects of post-SE brain injury. Besides these effects, SAR is a potent inhibitor of the ATP-binding cassette transporter subfamily B member 1 (ABCB1/MDR1/P-glycoprotein) (Liu et al., 2013), a major multidrug efflux transporter implicated in drug-resistant epilepsy (Löscher et al., 2020). Therefore, combining SAR with conventional antiseizure drugs which target neuronal excitability, may yield synergistic outcomes by maintaining effective therapeutic concentrations of antiseizure drugs in the brain, and warrants further investigations. The SAR dose used in this study translates to 195 mg human equivalent dose, which is higher than the 175 mg human equivalent maximum tolerable dose from cancer studies (Baselga et al., 2010; Fujisaka et al., 2013). Future ongoing studies in our laboratory will investigate the effects of SAR treatment at lower dosing regimen for a longer duration, instead of for a week, to determine an optimized therapeutic dose.

In conclusion, the goal of this study was to examine the effects of SFK inhibition on early epileptogenesis markers using SAR in a mixed-sex cohort of adult rats in the rat KA model of TLE. Our results show that KA-induced SE results in similar degree of brain pathologies in male and female rats. SAR treatment shows a similar degree of rescue in pathology across sexes. SAR treatment at 20 mg/kg once daily for 7 days mitigated the KA-induced microgliosis and neurodegeneration, with a limited effect on reactive microgliosis. These results indicate that SAR, despite a week’s treatment, mitigated some of the key markers of neurodegeneration and gliosis in both sexes. Future studies will examine the long-term treatment effects of SAR in preventing or modifying the comorbidities associated with post-SE brain injury.

## Data availability statement

The original contributions presented in this study are included in this article/Supplementary material, further inquiries can be directed to the corresponding author.

## Ethics statement

The animal study was approved by the Iowa State University Institutional Animal Care and Use Committee. The study was conducted in accordance with the local legislation and institutional requirements.

## Author contributions

NR: Data curation, Formal analysis, Investigation, Methodology, Validation, Visualization, Writing – original draft. MP: Data curation, Investigation, Validation, Writing – review and editing. CM: Investigation, Validation, Writing – review and editing. AA: Investigation, Writing – review and editing, Formal analysis. TT: Writing – review and editing, Conceptualization, Funding acquisition, Methodology, Project administration, Supervision.

## Footnotes

1
https://rdrr.io/bioc/maxcontrastprojection

2
https://imagej.net/plugins/analyze-skeleton
