# Supplementary material for: The effects of Src tyrosine kinase inhibitor, saracatinib, on the markers of epileptogenesis in a mixed-sex cohort of adult rats in the kainic acid model of epilepsy
Source: Front Mol Neurosci. 2023 Nov 9;16:1294514. doi: 10.3389/fnmol.2023.1294514 (PMC10665569; doi:10.3389/fnmol.2023.1294514)
Supplement: Supplementary file 1 [file Data_Sheet_1.docx]

Supplementary Material

The effects of Src Tyrosine Kinase Inhibitor, Saracatinib, on the markers of epileptogenesis in a mixed-sex cohort of adult rats in the kainic acid model of epilepsy

**Nikhil S. Rao, Marson Putra, Christina Meyer, Aida Almanza and Thimmasettappa Thippeswamy***

*** Correspondence:** Corresponding Author: tswamy@iastate.edu

# Tables

## Table 1. Antibodies used for IHC

| **Primary Antibody** | **Source** | **Catalogue number** | **Dilution factor** |
| --- | --- | --- | --- |
| Anti-NeuN (rabbit) | EMD Millipore | ABN78 | 1:200 |
| Anti-IBA1 (goat) | Abcam | Ab5076 | 1:300 |
| Anti-GFAP (mouse) | Sigma Aldrich | G3893 | 1:300 |
| Anti-pSrc (rabbit) | Invitrogen | 44-660G | 1:100 |
| Anti-CD68 (rabbit) | Abcam | Ab125212 | 1:300 |
| Anti-C3 (rat) | Novus Biologicals | NB200-540 | 1:80 |
| Anti-parvalbumin (Rabbit) | Abcam | Ab11427 | 1:1000 |
| **Secondary Antibody** | **Source** | **Catalogue number** | **Dilution factor** |
| AMCA blue streptavidin | Jackson ImmunoResearch | 016-150-084 | 1:80 |
| Cy3™-conjugated Streptavidin | Jackson ImmunoResearch | 016-160-084 | 1:80 |
| Biotinylated Donkey anti-Goat | Jackson ImmunoResearch | 711-295-152 | 1:100 |
| Biotinylated Donkey anti-Rat | Jackson ImmunoResearch | 712-065-153 | 1:100 |
| AlexaFluor 488 anti-mouse | Jackson ImmunoResearch | 115-545-003 | 1:300 |
| AlexaFluor 488 anti-rabbit | Jackson ImmunoResearch | 711-545-152 | 1:200 |
| Rhodamine Red X anti-rabbit | Jackson ImmunoResearch | 111-295-144 | 1:300 |
| Rhodamine Red X anti-goat | Jackson ImmunoResearch | 705-295-147 | 1:300 |

## Table 2. Mean ±SEM values & statistical tests for sex-interaction

| **Figure1** | **Proestrus** | **Estrus** | **Metestrus** | **Diestrus** | **Statistical**  **analysis** |  |  |
| --- | --- | --- | --- | --- | --- | --- | --- |
| C | 50.50 ± 15.50 | 57.33 ± 2.321 | 41.50 ± 11.15 | 47.40 ± 9.579 | One-way ANOVA (Holm-Sidak’s multiple comparisons test) | | |
|  | **KA + VEH** | **KA + SAR** |  |  |  |  |  |
| D | 8.47 ± 1.943 | 4.769 ± 1.428 |  |  | 2-way ANOVA (Sidak’s multiple comparison) | | |
| **Figure 2C** | **Control** | **KA + VEH** | **KA + SAR** | **Interaction effects P value** | **Statistical analysis** | | |
| DG | 38.22 ± 3.161 | 144.6 ± 12.21 | 108 ± 12.54 | 0.9770 | 2-way ANOVA (Tukey’s multiple comparison) | | |
| CA3 | 37.81 ± 7.145 | 200.9 ± 25.06 | 104.1 ± 15.35 | 0.2761 | 2-way ANOVA (Tukey’s multiple comparison) | | |
| CA1 | 38.3 ± 5.103 | 113 ± 13.40 | 85.29 ± 15.97 | 0.2155 | 2-way ANOVA (Tukey’s multiple comparison) | | |
| SUB | 37.26 ± 2.850 | 97.05 ± 14.01 | 78.06 ± 14.63 | 0.5380 | 2-way ANOVA (Tukey’s multiple comparison) | | |
| PIR | 48.22 ± 5.748 | 152.4 ± 20.97 | 121.7 ± 26.69 | 0.1498 | 2-way ANOVA (Tukey’s multiple comparison) | | |
| AMY | 56.06 ± 7.972 | 149.5 ± 20.02 | 82.35 ± 13.80 | 0.1568 | 2-way ANOVA (Tukey’s multiple comparison) | | |
| **Figure 2E** | **Control** | **KA + VEH** | **KA + SAR** | **Interaction effects P value** | **Statistical analysis** | | |
| DG | 20.18 ± 1.745 | 12.02 ± 0.8404 | 11.44 ± 0.8474 | 0.9824 | 2-way ANOVA (Tukey’s multiple comparison) | | |
| CA3 | 11.72 ± 1.292 | 6.692 ± 0.6742 | 6.80 ± 0.3833 | 0.7097 | 2-way ANOVA (Tukey’s multiple comparison) | | |
| CA1 | 6.937 ± 0.3830 | 4.950± 0.6290 | 5.596± 0.5930 | 0.3610 | 2-way ANOVA (Tukey’s multiple comparison) | | |
| SUB | 8.926 ± 0.8280 | 6.439 ± 0.4905 | 6.877 ± 0.7965 | 0.3415 | 2-way ANOVA (Tukey’s multiple comparison) | | |
| PIR | 10.64 ± 1.051 | 6.004 ± 1.130 | 5.627 ± 1.095 | 0.8414 | 2-way ANOVA (Tukey’s multiple comparison) | | |
| AMY | 10.56 ± 0.6480 | 8.001 ± 1.277 | 8.735 ± 0.7874 | 0.1476 | 2-way ANOVA (Tukey’s multiple comparison) | | |
| **Figure 3C** | **Control** | **KA + VEH** | **KA + SAR** | **Interaction effects P value** | **Statistical analysis** | | |
| DG | 5.812 ± 0.6751 | 5.545 ± 0.5934 | 4.795 ± 0.4312 | 0.8421 | 2-way ANOVA (Tukey’s multiple comparison) | | |
| CA3 | 5.750 ± 0.7148 | 6.333 ± 0.9503 | 5.5 ± 0.7917 | 0.7621 | 2-way ANOVA (Tukey’s multiple comparison) | | |
| CA1 | 6.458 ± 0.5154 | 3.485 ± 0.5325 | 2.667 ± 0.2799 | 0.6881 | 2-way ANOVA (Tukey’s multiple comparison) | | |
| SUB | 6.458 ± 1.493 | 5.985 ± 0.8820 | 3.923 ± 0.6992 | 0.0614 | 2-way ANOVA (Tukey’s multiple comparison) | | |
| PIR | 4.958 ± 1.066 | 2.242 ± 0.5651 | 1.974 ± 0.8460 | 0.5505 | 2-way ANOVA (Tukey’s multiple comparison) | | |
| AMY | 10.62 ± 1.331 | 4.939 ± 1.211 | 3.949 ± 0.7978 | 0.1279 | 2-way ANOVA (Tukey’s multiple comparison) | | |
| **Figure 4C** | **Control** | **KA + VEH** | **KA + SAR** | **Interaction effects P value** | **Statistical analysis** | | |
| DG | 1.669 ± 0.8551 | 40.25 ± 8.291 | 22.62 ± 5.431 | 0.8167 | 2-way ANOVA (Tukey’s multiple comparison) | | |
| CA3 | 1.875 ± 1.231 | 31.38 ± 7.492 | 15.73 ± 4.408 | 0.9444 | 2-way ANOVA (Tukey’s multiple comparison) | | |
| CA1 | 1.049 ± 0.5603 | 9.382 ± 5.547 | 9.190 ± 3.487 | 0.8434 | 2-way ANOVA (Tukey’s multiple comparison) | | |
| SUB | 0.8638 ± 0.5718 | 9.083 ± 3.747 | 5.547 ± 2.547 | 0.8832 | 2-way ANOVA (Tukey’s multiple comparison) | | |
| PIR | 2.525 ± 1.832 | 26.49 ± 6.619 | 12.50 ± 2.087 | 0.5501 | 2-way ANOVA (Tukey’s multiple comparison) | | |
| AMY | 3.450 ± 1.697 | 36.18 ± 7.498 | 29.44 ± 4.437 | 0.8902 | 2-way ANOVA (Tukey’s multiple comparison) | | |
| **Figure 5D** | **Control** | **KA + VEH** | **KA + SAR** | **Interaction effects P value** | **Statistical analysis** | | |
| DG | 113.2 ± 15.06 | 252.9 ± 32.54 | 274.7 ± 14.39 | 0.8293 | 2-way ANOVA (Tukey’s multiple comparison) | | |
| CA3 | 91.81 ± 10.56 | 229.3 ± 22.84 | 230.6 ± 20.38 | 0.0523 | 2-way ANOVA (Tukey’s multiple comparison) | | |
| CA1 | 106.5 ± 9.124 | 239.6 ± 15.35 | 215.6 ± 12.29 | 0.6074 | 2-way ANOVA (Tukey’s multiple comparison) | | |
| SUB | 105.4 ± 12.81 | 238.7 ± 15 | 245.5 ± 15.39 | 0.3923 | 2-way ANOVA (Tukey’s multiple comparison) | | |
| PIR | 106.6 ± 14.48 | 271 ± 18.57 | 237.1 ± 19.25 | 0.5774 | 2-way ANOVA (Tukey’s multiple comparison) | | |
| AMY | 122.6 ± 9.138 | 246 ± 26.21 | 263.8 ± 38.23 | 0.2216 | 2-way ANOVA (Tukey’s multiple comparison) | | |
| **Figure 5E** | **Control** | **KA + VEH** | **KA + SAR** | **Interaction effects P value** | **Statistical analysis** | | |
| DG | 0.04167 ± 0.04167 | 4.545 ± 1.838 | 4.295 ± 2.691 | 0.9302 | 2-way ANOVA (Tukey’s multiple comparison) | | |
| CA3 | 0.04167 ± 0.04167 | 2.909 ± 1.235 | 1.872 ± 1.077 | 0.5257 | 2-way ANOVA (Tukey’s multiple comparison) | | |
| CA1 | 0.1042 ± 0.0699 | 7.515 ± 1.951 | 8.436 ± 2.386 | 0.6941 | 2-way ANOVA (Tukey’s multiple comparison) | | |
| SUB | 0.0625 ± 0.0625 | 5.273 ± 1.565 | 4.282 ± 1.587 | 0.9833 | 2-way ANOVA (Tukey’s multiple comparison) | | |
| PIR | 0 ± 0 | 16 ± 4.458 | 7.897 ± 1.993 | 0.8771 | 2-way ANOVA (Tukey’s multiple comparison) | | |
| AMY | 0 ± 0 | 12.06 ± 2.977 | 7.269 ± 3.208 | 0.9314 | 2-way ANOVA (Tukey’s multiple comparison) | | |
| **Figure 6C** | **Control** | **KA + VEH** | **KA + SAR** | **Interaction effects P value** | **Statistical analysis** | | |
| DG | 1.708 ± 0.5650 | 44.75 ± 14.46 | 45.64 ± 15.61 | 0.6317 | 2-way ANOVA (Tukey’s multiple comparison) | | |
| CA3 | 0.8954 ± 0.3577 | 56.61 ± 18.01 | 44.02 ± 12.10 | 0.1962 | 2-way ANOVA (Tukey’s multiple comparison) | | |
| CA1 | 1.686 ± 0.3284 | 20.84 ± 7.308 | 32.23 ± 8.626 | 0.6105 | 2-way ANOVA (Tukey’s multiple comparison) | | |
| SUB | 1.875 ± 0.6297 | 31.11 ± 12.31 | 32.22 ± 9.557 | 0.9054 | 2-way ANOVA (Tukey’s multiple comparison) | | |
| PIR | 4.458 ± 0.9259 | 90.83 ± 38.89 | 139.6 ± 35.64 | 0.5529 | 2-way ANOVA (Tukey’s multiple comparison) | | |
| AMY | 3.040 ± 0.7904 | 61.39 ± 19.17 | 110.9 ± 28.36 | 0.9005 | 2-way ANOVA (Tukey’s multiple comparison) | | |
| **Figure 7** | **Control** | **KA + VEH** | **KA + SAR** | **Interaction effects P value** | **Statistical analysis** | | |
| B | 45.59± 4.515 | 14.01± 1.004 | 14.33± 0.9858 | 0.9476 | 2-way ANOVA (Tukey’s multiple comparison) | | |
| C | 41.28± 4.719 | 10.53± 0.8667 | 10.87± 0.7686 | 0.9658 | 2-way ANOVA (Tukey’s multiple comparison) | | |
| D | 70.62± 10.16 | 14.56± 1.797 | 15.02± 1.354 | 0.8374 | 2-way ANOVA (Tukey’s multiple comparison) | | |
| E | 48.21± 3.421 | 62.75± 7.118 | 51.98± 2.893 | 0.9008 | 2-way ANOVA (Tukey’s multiple comparison) | | |
| **Figure 8C** | **Control** | **KA + VEH** | **KA + SAR** | **Interaction effects P value** | **Statistical analysis** | | |
| DG | 1.854± 1.082 | 22.30± 3.239 | 17.06± 4 | 0.6404 | 2-way ANOVA (Tukey’s multiple comparison) | | |
| CA3 | 1.104± 0.4848 | 28.76± 5.126 | 18.55± 3.227 | 0.9027 | 2-way ANOVA (Tukey’s multiple comparison) | | |
| CA1 | 0.5417± 0.1775 | 28.27± 4.375 | 26.86± 5.568 | 0.9469 | 2-way ANOVA (Tukey’s multiple comparison) | | |
| SUB | 1.021± 0.4984 | 21.29± 4.015 | 16.90± 3.960 | 0.4107 | 2-way ANOVA (Tukey’s multiple comparison) | | |
| PIR | 1.229± 0.5695 | 17.09± 2.744 | 18.27± 2.951 | 0.4127 | 2-way ANOVA (Tukey’s multiple comparison) | | |
| AMY | 1.50± 0.5229 | 37.91± 6.367 | 28.96± 3.259 | 0.8055 | 2-way ANOVA (Tukey’s multiple comparison) | | |

# Supplementary Figures


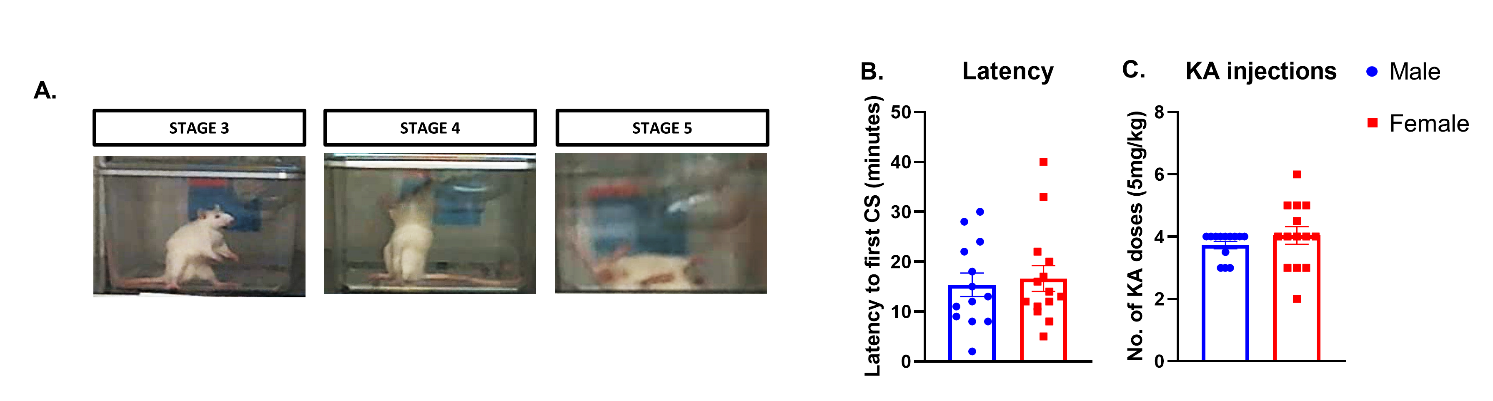


**Supplementary Figure 1.** (A) Representative convulsive seizure stages (stages 3-5) captured during Racine scoring. (B) Latency to the onset of the first convulsive seizure following the last intraperitoneal kainic acid (KA) injection. (C) The number of KA injections required to induce status epilepticus in both sexes. (B, C) Mann-Whitney test.


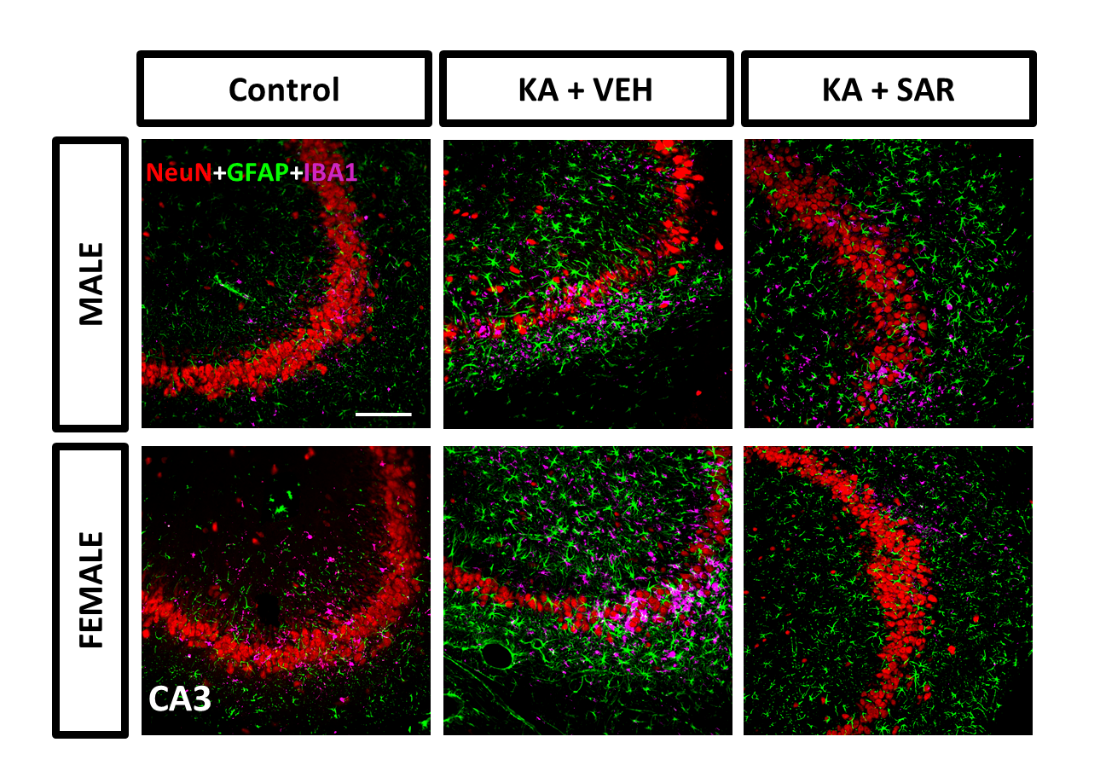


**Supplementary Figure 2.** Representative merged images of the CA3 region of the hippocampus stained for Neurons (NeuN, red), microglia (IBA1, pink) and astrocytes (GFAP, green) in male and female rats from the different treatment groups. Scale bar 100µm (all).


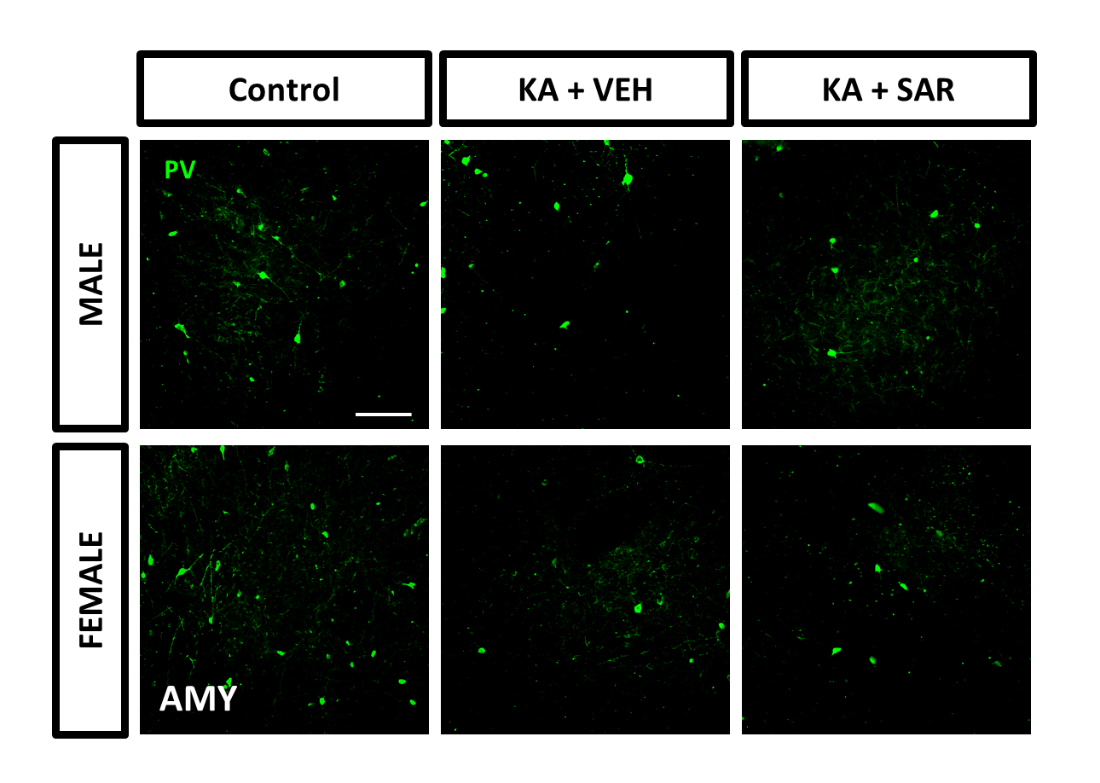


**Supplementary Figure 3.** Representative images of parvalbumin (PV) positive inhibitory interneurons from the amygdala of male and female rats from the different treatment groups. Scale bar 100µm (all).


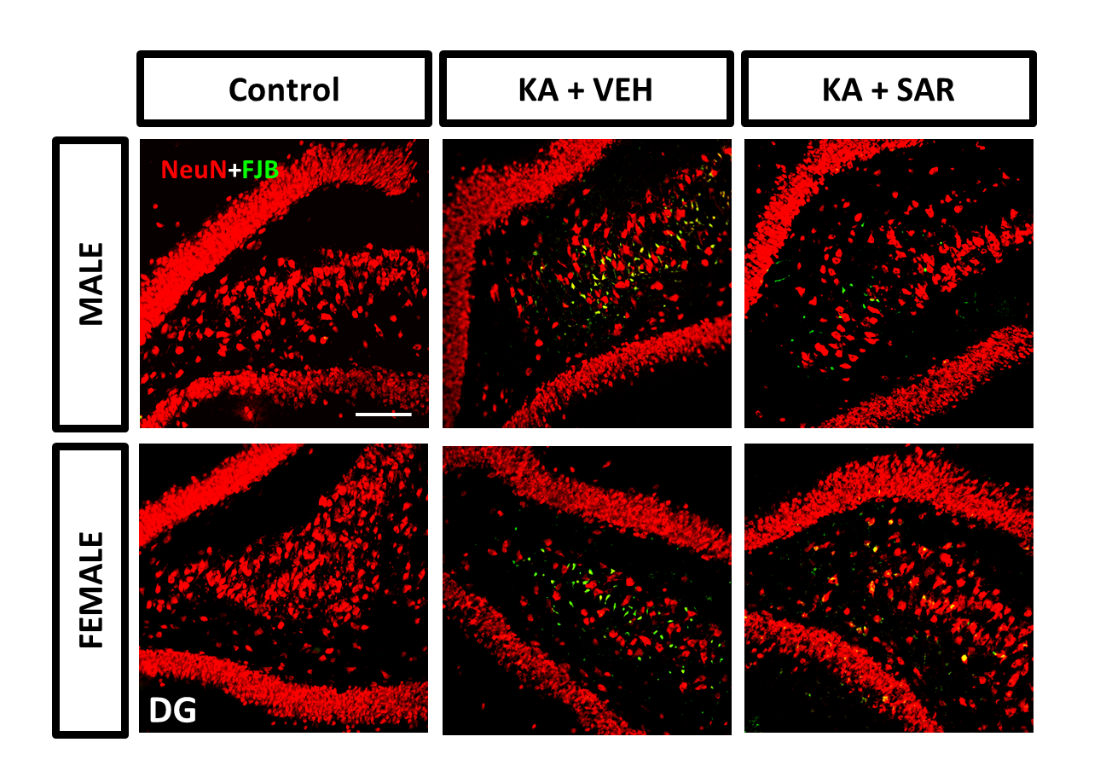


**Supplementary Figure 4.** Representative images of neurons (NeuN) co-stained with Fluorojade B (FJB) in the dentate gyrus of the hippocampus in male and female rats from the different treatment groups. Scale bar 100µm (all).


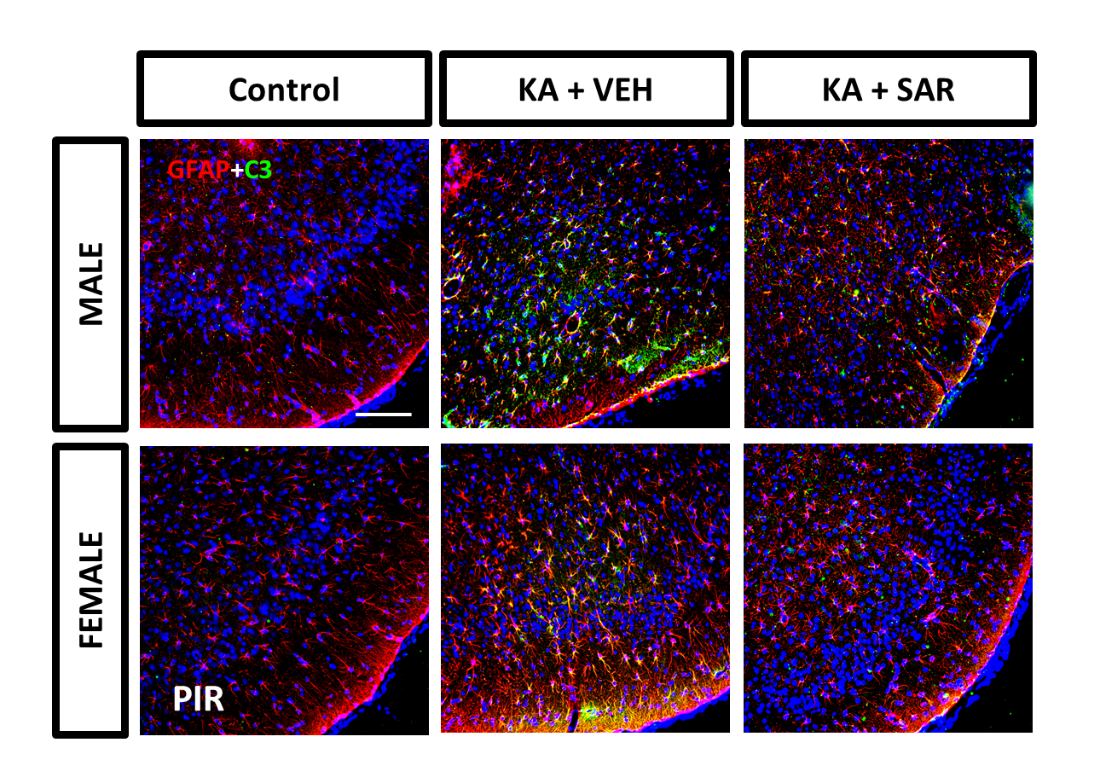


**Supplementary Figure 5.** Representative images of astrocytes (GFAP) co-stained with complement 3 (C3) in the piriform cortex of male and female rats from the different treatment groups. Scale bar 100µm (all).


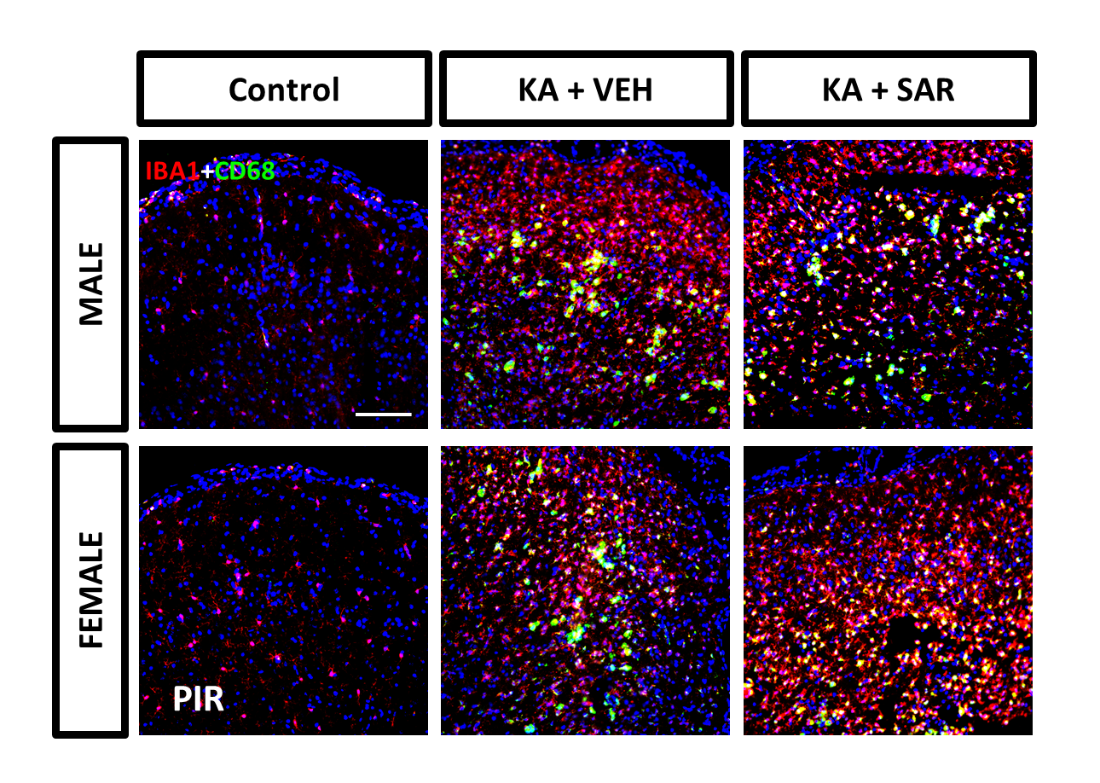


**Supplementary Figure 6.** Representative images of microglia (IBA1) co-stained with CD68 in the piriform cortex of male and female rats from the different treatment groups. Scale bar 100µm (all).


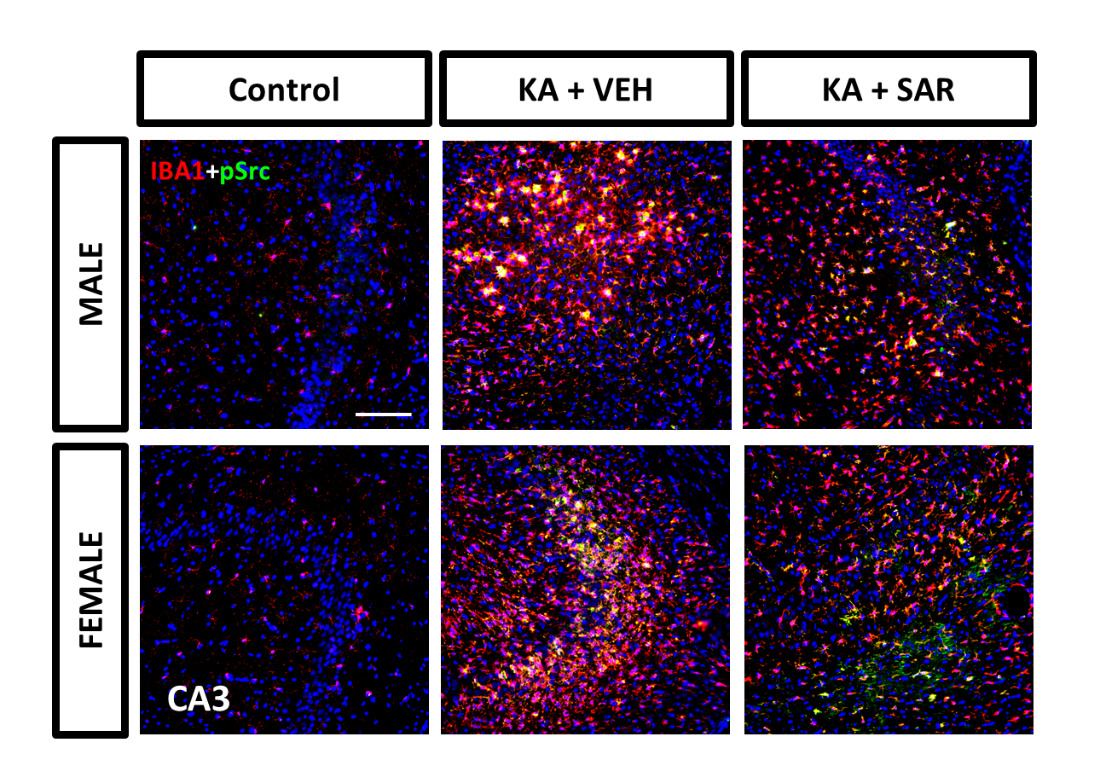


**Supplementary Figure 7.** Representative images of microglia (IBA1) co-stained with pSrc in the CA3 region of the hippocampus in male and female rats from the different treatment groups. Scale bar 100µm (all).
